# Melatonin Treatment Improves Renal Fibrosis via miR-4516/SIAH3/PINK1 Axis

**DOI:** 10.3390/cells10071682

**Published:** 2021-07-03

**Authors:** Yeo Min Yoon, Gyeongyun Go, Sungtae Yoon, Ji Ho Lim, Gaeun Lee, Jun Hee Lee, Sang Hun Lee

**Affiliations:** 1 Medical Science Research Institute, Soonchunhyang University Seoul Hospital, Seoul 04401, Korea; yoonboo15@naver.com; 2 Department of Biochemistry, College of Medicine, Soonchunhyang University, Cheonan 31151, Korea; ggy0227@naver.com (G.G.); wlenfl1@naver.com (J.H.L.); lun4905@naver.com (G.L.); 3 Department of Biochemistry, BK21FOUR Project2, College of Medicine, Soonchunhyang University, Cheonan 31151, Korea; 4 Stembio. Ltd., Entrepreneur 306, Soonchunhyang-ro 22, Sinchang-myeon, Asan 31538, Korea; sy2703@columbia.edu; 5 Institute of Tissue Regeneration Engineering (ITREN), Dankook University, Cheonan 31116, Korea; j-school@hanmail.net; 6 Department of Nanobiomedical Science and BK21 PLUS NBM Global Research Center for Regenerative Medicine, Dankook University, Cheonan 31116, Korea; 7 Department of Oral Anatomy, College of Dentistry, Dankook University, Cheonan 31116, Korea; 8 Cell & Matter Institute, Dankook University, Cheonan 31116, Korea

**Keywords:** chronic kidney disease, melatonin, miR-4516, mitophagy, PINK1, renal fibrosis, SIAH3

## Abstract

Dysregulation in mitophagy, in addition to contributing to imbalance in the mitochondrial dynamic, has been implicated in the development of renal fibrosis and progression of chronic kidney disease (CKD). However, the current understanding of the precise mechanisms behind the pathogenic loss of mitophagy remains unclear for developing cures for CKD. We found that miR-4516 is downregulated and its target SIAH3, an E3 ubiquitin protein ligase that reduces PINK1 accumulation to damaged mitochondria, is upregulated in the renal cortex of CKD mice. Here, we demonstrated that melatonin injection induces miR-4516 expression and suppresses SIAH3, and promotes PINK1/Parkin-mediated mitophagy. Furthermore, we demonstrated that melatonin injection attenuates the pathological features of CKD by improving mitochondrial homeostasis. Our data supports that mitochondrial autophagy regulation by activating miR-4516/SIAH3/PINK1 mitophagy signaling axis can be a viable new strategy for treating CKD.

## 1. Introduction

Fibrosis is an excessive form of tissue injury repair, where wound healing becomes progressively uncontrolled and pathological [1]. Pathological fibrosis leads to multiple organ dysfunction [2] and is the key contributor to poor outcome in many illnesses including chronic kidney disease (CKD) [3]. In CKD, fibrosis in kidney tissues negatively impacts renal function, and allows uremic toxins to remain in systemic circulation [4], leading to other conditions like myocardial fibrosis and resultant cardiovascular disease that propels the high mortality in late stage CKD patients [5]. Nonetheless, developing an effective treatment for reversing the pathological fibrosis in kidney has remained challenging [6]. Recent studies have suggested that dysregulation of mitochondrial quality control (MQC) mechanisms such as mitochondrial fission, fusion, and autophagy, plays a critical role in the progression of renal fibrosis [7]. As observation of abnormal mitochondria accumulation in renal tubular cells is a characteristic of CKD [8], specific interventions to promote MQC processes to counter the fibrosis progression has emerged as a promising therapeutic strategy [9,10].

PINK1/Parkin-mediated mitophagy is one of the major pathways for clearance of unhealthy mitochondria. In damaged mitochondria, PTEN-induced putative kinase protein 1 (PINK1), which is rapidly and constitutively degraded in steady state conditions in healthy mitochondria [11], starts to accumulate in the outer membrane to recruit Parkin, an E3 ubiquitin ligase, to initiate mitophagy [12]. Downregulation of mitophagy regulators downstream of PINK1, namely mitofusin-1 (MFN1) and Parkin, and the consequential accumulation of dysfunctional mitochondria has been confirmed for both experimental models and human kidney fibrosis [13].

Melatonin (N-acetyl-5-methoxy tryptamine), an endocrine hormone released by the pineal gland, is capable of promoting mitophagy for clearing unhealthy mitochondria [14], showing potential for ameliorating fibrosis [15]. We have previously demonstrated that melatonin can suppress ischemia-induced fibrosis in experimental models [16], and provide protective effects against renal cortical fibrosis by increasing the expression of miR-4516, a microRNA downregulated in the kidney cortex under CKD condition [17]. Nonetheless, the molecular mechanism and downstream effector of therapeutic effects of melatonin has yet to be investigated.

In this study, we show that melatonin-induced miR-4516 upregulation inhibits the expression of SIAH3, a known E3 ubiquitin protein ligase that reduces PINK1 accumulation to damaged mitochondria. Screening of microRNA target databases has identified SIAH3 as a potential downstream target for miR-4516 with respect to PINK1/Parkin-mediated mitophagy. We observed an increase in miR-4516 enhanced mitophagy in human proximal tubule (TH1) cells under *p*-cresol exposure by downregulating the SIAH3 expression. Short hairpin RNA-mediated knockdown of SIAH3 in TH1 cells showed enhanced activation of PINK1/Parkin-mediated mitophagy, which is identical to the outcome achieved with melatonin treatment. In CKD mouse models, intraperitoneal injection of melatonin reduced the number of abnormal mitochondria and ameliorated glomerulus swelling. This was consistent with the decreased in creatinine and blood urea nitrogen levels, which are clinical indicators for improved renal function. Our results suggest that mitophagy-defective chronic kidney injury can be attenuated or reversed by activating the miR-4516/SIAH3/PINK1 mitophagy signaling with melatonin. Thus, targeting this novel signaling mechanism could be a novel strategy to develop effective clinical treatments of renal fibrosis.

## 2. Materials and Methods

### 2.1. Culture of Human Proximal Tubular Epithelial (TH1) Cells

Human proximal tubular epithelial (TH1) cells (purchased from American Type Culture Collection, Manassas, VA, USA) were cultured with minimum essential medium supplemented with 10% (*v*/*v*) fetal bovine serum and 100 U/mL penicillin/streptomycin (all from Gibco BRL, Gaithersburg, MD, USA) in a humidified incubator at 5% CO_2_, 37 °C condition. In order to simulate the CKD condition, TH1 cells were exposed to 0.5 mM *p*-Cresol for 72 h. To examine the protective effect of melatonin, TH1 cells were pretreated with 1 μM melatonin for 24 h. In another experiment, when cells reached 70–80% confluence, they were transfected with 50 nM miR-4516 inhibitor (Applied Biological Materials, Richmond, VA, USA) or sh-SIAH3 lentiviral particle (OriGene, Rockville, MD, USA). 

### 2.2. Mitochondrial Fraction Preparation

For evaluating mitochondrial functions, we harvested TH1 cells and isolated their mitochondria using mitochondria fraction kit (Thermo Fisher Scientific, Waltham, MA, USA). Isolated mitochondria were subsequently incubated for 10 min on ice. The cell lysates with isolation buffer were centrifuged at 700× *g* for 10 min at 4 °C. Supernatant was collected and centrifuged again at 12,000× *g* for 15 min at 4 °C. After washing with fraction buffer, the residual pellet was lysed with RIPA lysis buffer and then centrifuged at 12,000× *g* for 30 min at 4 °C.

### 2.3. Quantification of miRNA

Total RNA was isolated from TH1 cells or kidney cortex tissue preparations using mirVana^TM^ miRNA isolation kit (Thermo Fisher Scientific). cDNA of the extracted miRNA was synthesized with poly(A) polymerase tailing (Applied Biological Materials; abm, Richmond, BC, Canada). Quantitative real-time polymerase chain reaction (qRT-PCR) was performed with the TaqManTM Small RNA Assay (Thermo Fisher Scientific). Expression of miRNAs was normalized with β-actin as a control. 

### 2.4. Transmission Electron Microscopy

Samples from mice kidney cortex (approximately 1 mm^3^) or TH1 cells (~107 cells) were fixed with Karnovsky’s fixative (0.1 M sodium cacodylate buffer with 2% paraformaldehyde and 2.5% glutaraldehyde, pH 7.3). After washing 3 times with 0.05 M sodium cacodylate buffer, postfix step was carried out in 2% osmium tetroxide and 0.01 M cacodylate buffer. Fixed samples were dehydrated in 30~100% ethanol and embedded in epoxy resin. Ultrathin sections (~70 nm) were cut with an ultramicrotome (Leica, Wetzlar, Germany), and mounted on a copper grid. The section samples were detected with a transmission electron microscope (Field Electron and Ion Company, Protland, OR, USA). Morphometric analyses (the number of mitochondria per cell and mitochondrial size) were performed in ImageJ software (NIH; version 1.43). Mitochondrial size and number of abnormal mitochondria were examined for at least 50–100 individual mitochondria from different cells and renal cortex for each experimental group at 6300× magnification.

### 2.5. Western Blotting

The whole cell lysates, cytosol fraction lysates, or mitochondrial fraction lysates were prepared from TH1 cells and renal cortex tissue. Protein concentration was determined by employing the BCA method, and 30 μg of protein per sample was loaded for western blotting. Electrophoresis in 8–12% sodium dodecyl sulfate-polyacrylamide gel was followed by a transfer to polyvinylidene difluoride membranes. The blots were washed with TBST (10 mM Tris-HCl, 150 mM NaCl, 0.05% Tween-20) and blocked with 5% skim milk for 1 h at room temperature. Subsequently, we incubated the blot with the appropriate primary antibodies: against PINK1 (Santa Cruz Biotechnology, Dallas, TX, USA, sc-517353), SIAH3 (Novus, Littleton, CT, USA, NBP2-83524), LC3B (Novus, NB100-2220), P62/SQSTM1 (Novus, NBP1-48320), p-DRP1 (Ser637) (Cell Signaling Technology, #4867S), DRP1 (Santa Cruz Biotechnology, sc-271583), MFN1 (Santa Cruz Biotechnology, sc-166644), OPA1 (Novus, NBP1-71656), ubiquitin (Novus, NB300-130), VDAC1 (Novus, NB100-695), α-tubulin (Santa Cruz Biotechnology, sc-8035), and β-actin (Santa Cruz Biotechnology, sc-47778). Then, the membranes were washed again and incubated for detection with either goat anti-rabbit IgG or goat anti-mouse IgG secondary antibodies (Cell signaling, Danvers, MA, USA). The bands were detected by enhanced chemiluminescence (Amersham Pharmacia Biotech, Little Chalfont, UK).

### 2.6. Immunoprecipitation

Protein A/G PLUS-Agarose Immunoprecipitation Reagent (Santa Cruz Biotechnology) were mixed with anti-PINK1 antibodies (Santa Cruz Biotechnology) and incubated for 4 h at 4 °C. TH1 cells were lysed with lysis buffer (1% Triton X-100 in 50 mM Tris-HCl (pH 7.4), 150 mM NaCl, 5 mM EDTA, 2 mM Na_3_VO_4_, 2.5 mM Na_4_PO_7_, 100 mM NaF, and protease inhibitors), and a total of 300 μg of protein lysates were incubated with Agarose mixture for 12 h at 4 °C. The beads were washed four times, and the bound protein was released from beads by boiling in SDS-PAGE sample buffer for 7 min. The samples were analyzed by western blot with anti-ubiquitin and anti-SIAH3 antibodies. 

### 2.7. OCR Measurements and Calculation of Bioenergetic Parameters

The XF96 Extracellular Flux Analyzer (Agilent Technologies, Santa Clara, CA, USA) was used to monitor mitochondrial OCR. TH1 cells were seeded in a XF96 cell culture plate at 45,000 cells/well in growth media and incubated for 24 h in humidified atmosphere at 37 °C and 5% CO_2_. Each well was washed with XF assay medium (Agilent Technologies) with 25 mM glucose, and then incubated at 37 °C in non-CO_2_ for 30 min. OCR was measured every 7 min (after mixing for 3 min, waiting for 2 min, and measurement for 2 min), three times after injection of the respective compounds (1.5 μM oligomycin, 1 μM FCCP, and 0.5 μM Rotenone + Antimycin A), using Seahorse Wave Desktop Software (Agilent Technologies). Bioenergetic parameters were calculated from obtained OCR data; Basal mitochondrial OCR was derived by subtracting non-mitochondrial OCR (remaining COR after antimycin A addition) from baseline OCR prior to oligomycin treatment. Maximal respiration OCR was stimulated by FCCP addition, and OCR attributed to proton leak was calculated as the difference between OCR following inhibition with oligomycin A and OCR following inhibition with antimycin A treatment. ATP production was derived as the difference between basal and antimycin A-inhibited OCR. Spare Respiratory Capacity OCR was calculated as the difference between OCR following Oligomycin A inhibition and OCR following FCCP.

### 2.8. Fluorescence Staining for Flow Cytometry

To measure autophagosome activity, mitochondrial ROS, and number of active mitochondria, TH1 cells were collected and stained with Green Detection Reagent in autophagy assay kit (abcam, ab139484) at 37 °C for 30 min, 10 μM MitoSOX™ (Thermo Fisher Scientific) at 37 °C for 15 min, or 100 nM tetramethylrhodamine ethyl ester (TMRE) (Thermo Fisher Scientific) at 37 °C for 15 min for respective purposes. After washing with PBS, the sample cells were resuspended in 500 μL of PBS. The positive cells were detected using flow cytometry (Sysmex, Kobe, Japan). The data were analyzed using FCS Express 5 software (DeNovo Software, Los Angeles, CA, USA).

### 2.9. CKD Mouse Model

Eight-week-old male BALB/c mice were fed an adenine-containing diet (0.25% adenine) for 3 weeks, and body weights were measured every week. The mice were randomly distributed to one of four groups consisting of 10 mice in each group. After euthanasia, mice blood samples were stored at −80 °C for measuring BUN (MYBioSource, San Diego, CA, USA) and creatinine level (Crystal Chem, Elk Grove Village, IL, USA). In order to analyze the kidney-protecting effect of melatonin, each group was administered with either PBS, melatonin (0.2 mg/kg), or melatonin with miR-4516 inhibitor (300 nM) via intraperitoneal injection after the first week (after 7 days) of adenine feeding. Each group received 2 injections per week (every 3–4 days), a total of 4 injections for 2 weeks. The results were compared with healthy control. BUN and creatinine levels were measured. The kidneys were retained for further histological analysis.

### 2.10. Hematoxylin and Eosin (H&E), and Immunofluorescence Staining

After feeding 0.25% adenine for 3 weeks, mice kidney tissues were removed and fixed with 4% paraformaldehyde (Sigma Aldrich, MO, USA) and embedded in paraffin. The samples were stained with H&E in kidney tissues to determine histopathological features. Immunofluorescence staining was performed with primary antibodies against LAMP1 (Novus, NBP2-52721) and COX4 (Novus, NB110-39115), followed by secondary antibodies conjugated with Alexa Fluor 488 or 594 (Thermo Fisher Scientific). Nuclei were stained with 4′,6-diaminido-2-phenylindol (DAPI; Sigma), and the immune-stained samples were examined with confocal microscopy (Olympus, Tokyo, Japan).

### 2.11. Immunohistochemical Staining

The kidney sections were blocked in 3% hydrogen peroxide at RT for 15 min. The samples were then stained with primary rabbit anti-SIAH3 (1:500 dilution) antibody at RT for 2 h, followed by the HRP-linked anti-rabbit IgG secondary antibody (1:1500 dilution) at RT for 2 h. For visualizing antibodies and cell nuclei, a 3,3′-diaminobenzidine substrate kit (Vector Laboratories, Inc., Burlingame, CA, USA) and hematoxylin were used, respectively.

### 2.12. Ethics Statement

All animal experiments were approved and authorized by the Institutional Animal Care and Use Committee of Soonchunhyang University Seoul Hospital (IACUC2013-5, 16 February 2014). This study employed 8-week-old male male BALB/c mice (Biogenomics, Seoul, Korea), which were maintained on 12 h light followed by 12 h dark schedule at 25 °C as indicated by the guidelines established by the the National Research Council (NRC) Guidelines for the Care and Use of Laboratory Animals.

### 2.13. Statistical Analysis

Data are presented in the format of mean ± standard error of the mean (SEM). Statistical analysis between two groups was done by performing unpaired Student’s *t*-test. For multiple-group comparisons, one-way analysis of variance (ANOVA) was performed. In the comparisons for multiple groups (three or more), we employed the Bonferroni–Dunn test. Statistical significance was identified using *p* value cut-off of 0.05. All statistical analyses were carried out using SigmaPlot software (Systat Software, San Jose, CA, USA).

## 3. Results

### 3.1. The Kidney of CKD Mice Has Dysregulated Mitochondrial Dynamics and Mitophagy

**Figure 1:**
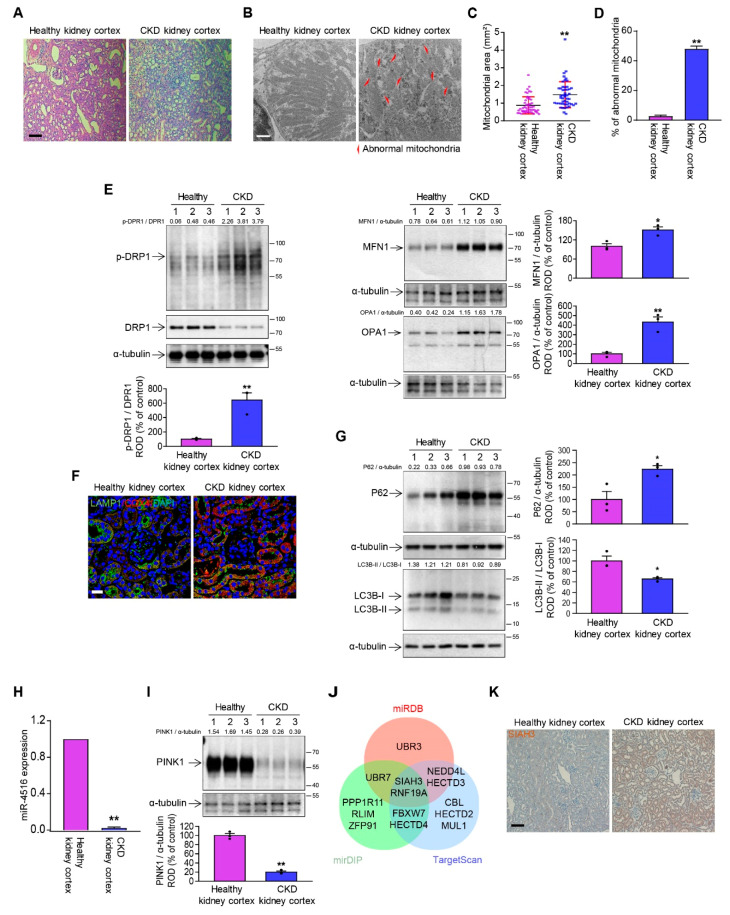
The miR-4516/SIAH3 pathway is involved in dysregulated mitophagy in fibrotic kidney of CKD mice. (**A**) H&E staining of kidney cortex of CKD mice with healthy control (scale bar = 100 μm). (**B**) Representative TEM images of mitochondria in renal cortex of healthy and CKD mice (scale bar = 1 μm). (**C**,**D**) Quantification of mitochondrial size and number of abnormal mitochondria in renal cortex of healthy and CKD mice (*n* = 3). (**E**) Expression of p-DRP1 (S637), DRP1, MFN1, and OPA1 in renal cortex of CKD mice with healthy control. (**F**) Immunofluorescence data for lysosomal marker LAMP-1 (green) and mitochondrial marker COX4 (red) in CKD renal cortex region with healthy control (scale bar = 20 μm). (**G**) Expression of P62 and LC3B in renal cortex of CKD mice with healthy control. Protein expression level were quantified by densitometry and normalized to α-tubulin levels (*n* = 3). (**H**) Level of miR-4516 in renal cortex of CKD mouse as measured by qRT-PCR. (**I**) Expression of PINK1 in renal cortex of CKD mice. (**J**) Potential target genes of miR-4516 related to E3 ubiquitin protein ligase as reported by three different microRNA databases. SIAH3 was suggested by all three databases as indicated at the intersection of the Venn diagram. (**K**) Representative IHC images for SIAH3 in renal cortex of CKD mice with healthy control. Protein expression level was quantified by densitometry and normalized to α-tubulin levels (*n* = 3). The values represent mean ± SEM, * *p*< 0.05, ** *p* < 0.01 compared with renal cortex of healthy mice. Statistical significance was assessed with unpaired Student’s *t*-test. The α-tubulin was used as Western blot loading control for whole tissue lysates.

To confirm whether mitochondrial dynamics and mitophagy are dysregulated in the kidney of CKD mice, CKD mice model were created in BALB/c mice by feeding with 0.25% adenine diet as previously described [18]. Pathogenesis of CKD was confirmed by observing increased fibrosis region and glomerulus size in H&E staining (Figure 1A). Many have reported on the role of dysregulated mitochondrial dynamics in CKD [7,19,20], and this was consistent with our TEM data showing abnormal mitochondrial morphology (Figure 1B). In addition to commonly reported mitochondrial fragmentation, we observed abnormally elongated mitochondria and an increase in both mitochondrial area and percentage of abnormal mitochondria (Figure 1C,D). To confirm that abnormal enlargement of mitochondria originated from dysregulation of mitochondrial dynamics, we investigated the expression of mitofusion marker proteins such as MFN1, optic atrophy 1 (OPA1), phosphor-dynamin-related protein 1 (p-DRP1(S637)), and mitofission maker protein DRP1 (unphosphorylated). The expression of all three mitofusion markers were excessively elevated in renal cortex of CKD mice compared to healthy control, while DRP1 showed noticeable downregulation (Figure 1E). This confirmed that the mitochondrial homeostasis has been disrupted. To determine whether mitophagy was decreased in renal cortex of CKD mice, immunofluorescence staining of kidney sections with lysosomal marker lysosomal-associated membrane protein 1 (LAMP-1) and mitochondrial marker cytochrome c oxidase subunit 4 (COX4) was performed. The results showed that co-localization of lysosomes and mitochondria was decreased in renal cortex of CKD mice compared to that of healthy mice suggesting attenuated mitophagy in the kidney of CKD mice (Figure 1F). In order to verify the downregulation of mitophagy in CKD mouse model, we examined the expression pattern of autophagosome markers such as P62 and microtubule-associated protein 1 light chain 3 (LC3). LC3 has a precursor form (LC3-I) and an active form (LC3-II) that promotes autophagy, and P62 is a pro-autophagy receptor and substrate [21]. High LC3-II/LC3-I ratio is associated with enhanced autophagy [22], while impaired autophagy leads to accumulation of P62 [23]. In our present study, we detected both depression of LC3-II/LC3-I ratio and accumulation of P62, as expected for the autophagy suppression in the kidney of CKD mice (Figure 1G).

### 3.2. miR-4516/SIAH3 Pathway Is Involved in Dysregulated Mitophagy

Our previous study showed that miR-4516 expression has a negative correlation with cytoskeleton reorganization and mitochondrial dysfunction in renal fibrosis, and promotion of miR-4516 with melatonin provides protective effects against CKD [17]. In the present study, we consistently observed that miR-4516 was markedly decreased in the CKD condition (Figure 1H). In addition, PINK1 expression was decreased in the renal cortex of CKD mice (Figure 1I). We searched microRNA databases to see if PINK1 is direct target of miR-4516. However, PINK1 has never been reported as a direct target of miR-4516. Therefore, we hypothesized that miR-4516 regulates the expression of PINK1 by regulating the expression of a E3 ubiquitin protein ligase. We searched three different microRNA databases (miRTarBase, mirDB, and TargetScan) to find miR-4516 target genes related to E3 ubiquitin protein ligase. SIAH3 and ring finger protein 19 A (RNF19A) were found as target genes of miR-4516 in three microRNA databases, of which SIAH3 had the highest target score (Figure 1J, Supplementary Tables S1–3). SIAH3 was found to be localized at mitochondria to inhibit PINK1 accumulation upon mitochondrial insult, reducing parkin-mediated mitophagy [24]. Our immunohistochemistry data showed increased SIAH3 expression in renal cortex of CKD mice (Figure 1K), supporting the idea that miR-4516 may suppress SIAH3-induced inhibition of PINK1/Parkin-mediated mitophagy in kidney injury. Taken together, these results suggest that miR-4516 is a novel upstream regulator of PINK1/Parkin pathway for mitophagy.

### 3.3. Melatonin-Induced miR-4516 Targets SIAH3 to Improve Mitophagy

**Figure 2:**
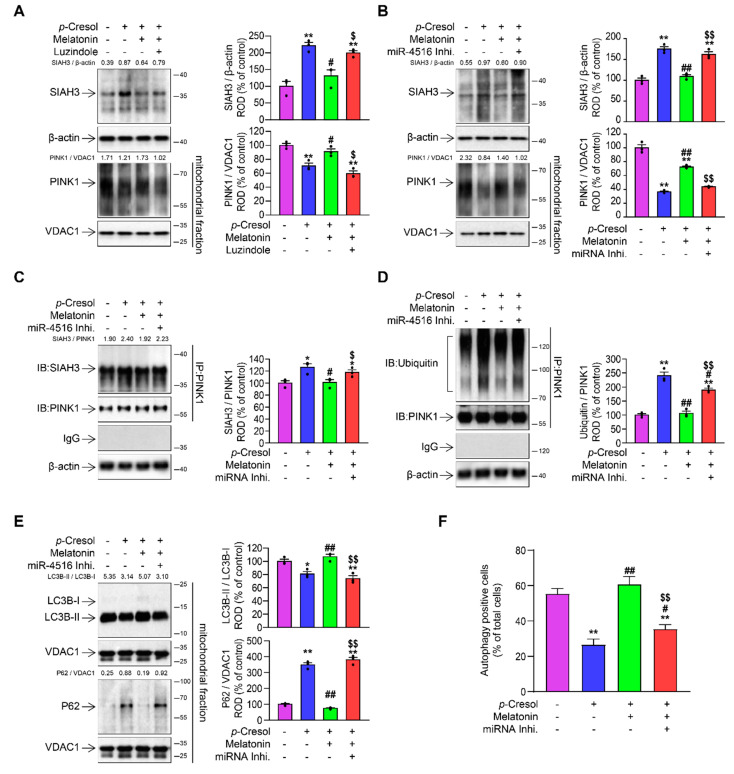
Melatonin-induced miR-4516 promotes PINK1/Parkin-mediated mitophagy by suppressing SIAH3. (**A**) Expression of SIAH3 and PINK1 in melatonin-treated TH1 cells (1 μM for 24 h) or pretreated with the luzindole (1 μM for 48 h) under *p*-Cresol exposure (0.5 mM for 72 h) (*n* = 3). Protein expression level was quantified by densitometry and normalized to β-actin or VDAC1 levels (*n* = 3). (**B**) Expression of SIAH3 and PINK1 in melatonin-treated TH1 cells (1 μM for 24 h) or pretreated with the miR-4516 inhibitor (50 nM for 48 h) under *p*-Cresol exposure (0.5 mM for 72 h) (*n* = 3). Protein expression level was quantified by densitometry and normalized to β-actin or VDAC1 levels (*n* = 3). (**C**) After immunoprecipitation against PINK1, the precipitates were analyzed by immunoblotting with SIAH3 antibody. Proteins signals were quantified by densitometry and were normalized to PINK1 levels (*n* = 3). (**D**) PINK1 immunoprecipitates were analyzed by immunoblotting with the ubiquitin antibody. (**E**) LC3B-II/LC3B-I ratio and P62 expression level was measured with immunoblotting. Protein expression was quantified by densitometry and normalized to VDAC1 levels (*n* = 3). (**F**) The percentage of autophagy positive cells in melatonin-treated TH1 cells (1 μM for 24 h) under *p*-Cresol exposure (0.5 mM for 72 h). TH1 cells were pretreated with the miR-4516 inhibitor (50 nM for 48 h) before melatonin treatment (*n* = 3). All reported values represent mean ± SEM, * *p* < 0.01, ** *p* < 0.01 versus control; ^#^ *p* < 0.05, ^##^ *p* < 0.01 versus *p*-Cresol exposure; ^$^ *p* < 0.05 ^$$^ *p* < 0.01 versus melatonin-treated cells under *p*-Cresol exposure. The β-actin or VDAC1 was used as Western blot loading control for whole cell lysates or mitochondrial fraction respectively.

To further validate the hypothesized miR-4516/SIAH3/PINK1 pathway, we created an in vitro model of CKD by treating human proximal epithelial tubular cells (TH1) with *p*-Cresol, a uremic toxin, to provide a CKD-like condition as previously described [17]. Treatment with *p*-Cresol (0.5 mM, 72 h) downregulated miR-4516 and PINK1 levels while increased SIAH3 expression level (Figure 2A) [17]. However, melatonin treatment of *p*-Cresol-treated TH1 cells increased miR-4516 levels and the translocation of PINK1 to mitochondria, while suppressing the expression of SIAH3 (Figure 2A) [17]. To confirm that miR-4516 upregulation was indeed induced by melatonin treatment, we pretreated TH1 cells with luzindole, a pharmacologic melatonin receptor inhibitor, before melatonin treatment. Inhibition of melatonin receptors effectively negated the rescuing effect of melatonin treatment, as it resulted in the decrease of miR-4516 and PINK1 expression and increase of SIAH3 expression (Figure 2A). We also questioned whether the observed expression patters of SIAH3 and its downstream effector PINK1 was achieved by a different regulatory pathway other than one involving miR-4516. Thus, we pretreated TH1 cells with miR-4516 inhibitor to see if silencing the miR-4516 would negate the regulatory effects of melatonin. The results showed that the effect of melatonin treatment on the translocation of PINK1 is inhibited by the knockdown of miR-4516 (Figure 2B). Although we confirmed that miR-4516 plays a role in SIAH3 downregulation and PINK1 upregulation, there remains the possibility that the promotion of PINK1 by miR-4516 and inhibition of SIAH3 could be independent of SIAH3/PINK1 interaction. Therefore, we checked the binding of SIAH3 and PINK1 through immunoprecipitation experiments. The immunoprecipitation of PINK1 showed weak SIAH3 binding (Figure 2C) and ubiquitination (Figure 2D) for melatonin treatment but knockdown of miR-4516 induced the strong SIAH3 binding and ubiquitination (Figure 2C,D). Furthermore, PINK1 expression was consistent with the expression of mitophagy markers, which demonstrated high LC3BII/LC3BI ratio and low P62 expression in melatonin-treated TH1 cells under *p*-Cresol exposure (Figure 2E). Detection of autophagy positive cells using flow cytometry showed consistent results that higher number of autophagy positive cells in melatonin-treated TH1 cells under *p*-Cresol exposure (Figure 2F). These results are in accordance with the current knowledge that the E3 ubiquitin protein ligase activity of SIAH3 marks PINK1 for proteasome degradation to suppress PINK1/Parkin-mediated mitophagy [24]. Furthermore, we checked the effect of shRNA-mediated knockdown of SIAH3 on PINK1/Parkin-mediated mitophagy in *p*-Cresol-treated TH1 cells. Successful execution of RNA interference against SIAH3 was confirmed with western blot showing decreased SIAH3 expression (Supplementary Figure S1A). The expression patterns for sh-SIAH3 treated TH1 cells under *p*-Cresol exposure was found to be identical to miR-4516 upregulated TH 1 cell by melatonin treatment in terms of upregulated PINK1, decreased SIAH3 binding of PINK1 for ubiquitination (Supplementary Figure S1A–C), and enhanced autophagy (Supplementary Figure S1D,E). Taken together, these results reveal that miR-4516 is involved in mitophagy regulation by regulating SIAH3 expression.

### 3.4. Melatonin-Induced miR-4516 Restore Mitochondrial Dysfunctions

**Figure 3:**
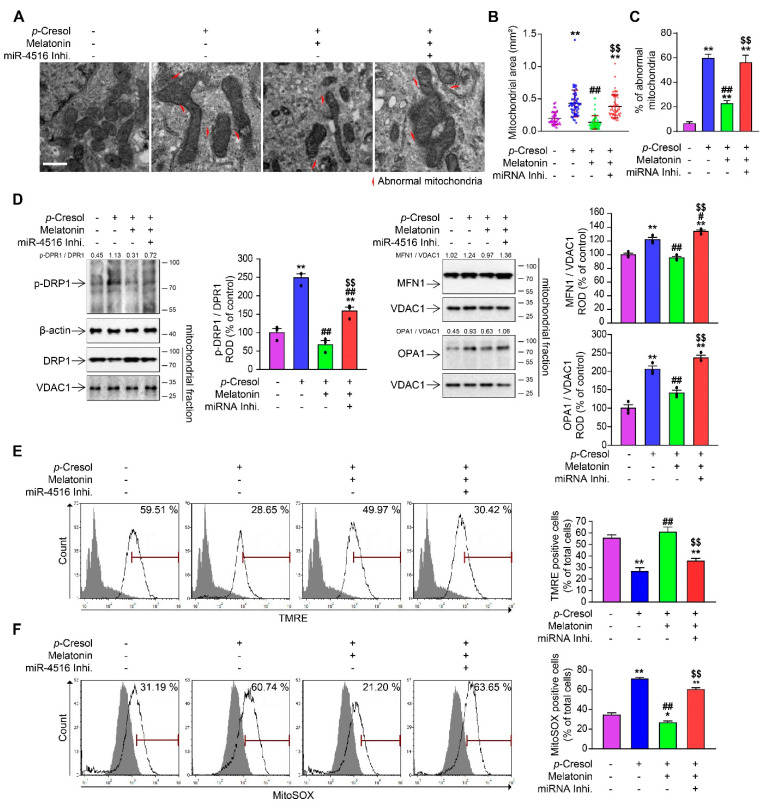
Melatonin-induced miR-4516 rescues abnormal mitochondrial functions. (**A**) Representative TEM images for TH1 cells either treated with *p*-Cresol alone, melatonin under *p*-Cresol exposure, or miR-4516 inhibitor (50 nM for 48 h) before melatonin treatment, compared with TH1 control (scare bar = 1 μm). (**B**,**C**) Measurement of mitochondrial area and number of abnormal mitochondria in each experimental group (*n* = 3). (**D**) The effects of melatonin on p-DRP1, DRP1, MFN1, and OPA1 were reversed with miR-4516 inhibitor. Protein expression level was detected using western blot, quantified by densitometry, and normalized to DRP1 or VDAC1 levels (*n* = 3) respectively. (**E**,**F**) Measurement of TMRE (**E**) and MitoSOX (**F**) positive cells for each group (*n* = 3). The values represent mean ± SEM, * *p* < 0.05, ** *p* < 0.01 versus control; ^#^ *p* < 0.05, ^##^ *p* < 0.01 versus *p*-Cresol exposure; ^$$^ *p* < 0.01 versus melatonin-treated cells in *p*-Cresol exposure. The β-actin or VDAC1 was used as Western blot loading control for whole cell lysates or mitochondrial fraction, respectively.

Subsequently, we examined the effect of melatonin-induced miR-4516 on abnormal mitochondrial dynamics and impaired mitochondrial functions, which are found in CKD. Examination of *p*-Cresol-treated TH1 cells with TEM showed abnormal mitochondrial morphology, with excessively elongated mitochondria, but this pathological feature was successfully reversed with melatonin-induced miR-4516 upregulation (Figure 3A). Quantification of the abnormal mitochondrial and cellular occupancy of mitochondria revealed that miR-4516 is capable of attenuating the accumulation of unhealthy mitochondria (Figure 3B,C). To confirm that the attenuation of excessive abnormal mitochondria in CKD has been accomplished via regulation of mitochondrial dynamics by miR-4516, we used immunoblotting assays to detect mitofusion and mitofission marker proteins. Upregulation of p-DRP1 (S637), MFN1, OPA1 and downregulation of DRP1 in *p*-Cresol-treated TH1 cells and miR-4516 inhibitor pretreated group suggested excessive mitofusion and insufficient mitofission at play, while melatonin treatment of TH1 cells restored the abnormal protein expressions (Figure 3D). In our previous study, we tested whether induction of miR-4516 can treat abnormal mitochondrial functions. Using XFe Extracellular Flux Analyzer, we found that oxygen consumption rate (OCR) of mitochondria in *p*-Cresol-treated TH1 cells showed impaired respiration, impaired ATP production, and compromised spare respiratory capacity [17]. Melatonin treatment to TH1 cells under *p*-Cresol exposure achieves partial rescue of mitochondrial functions. The additional use of miR-4516 inhibitor is capable of reversing this therapeutic effect [17]. Likewise, the number of TMRE positive cells was decreased after *p*-Cresol treatment, and yet melatonin-induced miR-4516 was able to restore them (Figure 3E). Production of mitochondrial ROS (mtROS) remained comparable to control only when miR-4516 remained active (Figure 3F). Thus, miR-4516 not only restores the normal morphology of mitochondria, but also their functions by the enhancement of mitophagy.

### 3.5. Melatonin Injection Ameliorates CKD via miR-4516/SIAH3/PINK1 Pathway

**Figure 4:**
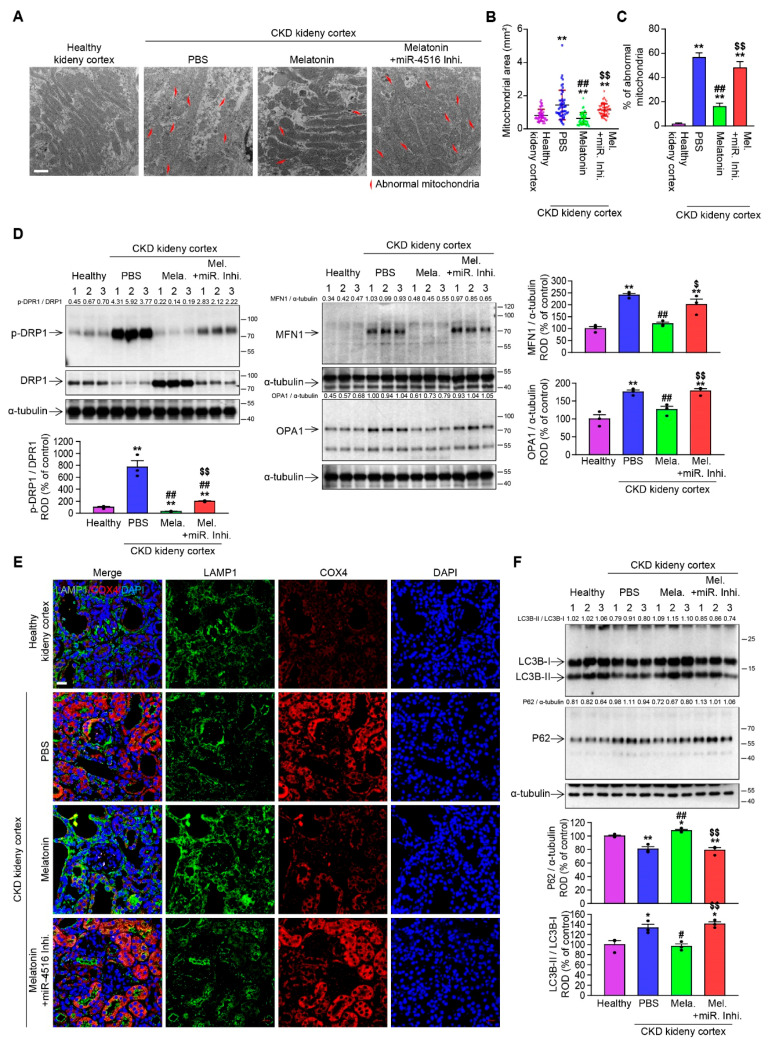
Melatonin-induced miR-4516 improves mitochondrial dynamics and enhances PINK1/Parkin-mediated mitophagy in the CKD mouse model. (**A**) Representative TEM images of mitochondria in renal cortex of CKD mice either treated with melatonin (0.2 mg/kg), or both melatonin and miR-4516 inhibitor (300 nM). Each group received two intraperitoneal injections per week (every 3–4 days)—a total of 4 injections for 2 weeks. All comparisons were made against healthy kidney control (scare bar = 1 μm) (**B**,**C**) Measurement of mitochondrial area and number of abnormal mitochondria in renal cortex of each groups (*n* = 3). (**D**) The expression of p-DRP1, DRP1, MFN1, and OPA1 in renal cortex of each group. Protein expression level were quantified by densitometry and normalized to DRP1 or α-tubulin levels (*n* = 3). (**E**) Immunofluorescence staining for LAMP-1 (green) and COX4 (red) in renal cortex of each group. Scare bar = 20 μm. (**F**) Expression of LC3B-II/LC3B-I ratio and P62 in renal cortex of each groups (*n* = 3). The values represent mean ± SEM, * *p* < 0.05, ** *p* < 0.01 versus healthy kidney cortex; ^#^ *p* < 0.05, ^##^ *p* < 0.01 versus PBS; ^$^ *p* < 0.05, ^$$^ *p* < 0.01 versus melatonin. The α-tubulin was used as Western blot loading control for whole tissue lysates.

**Figure 5:**
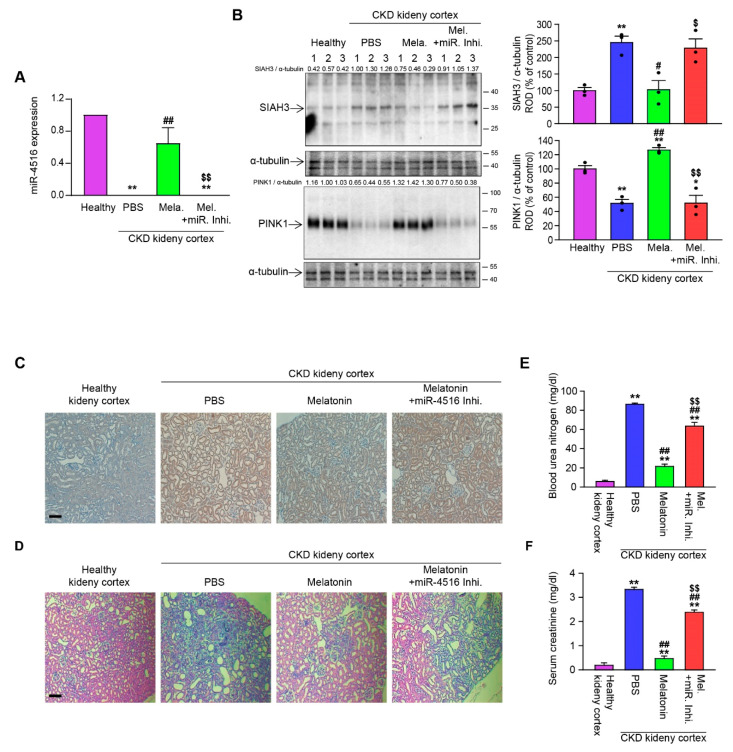
Melatonin injection ameliorates fibrotic CKD and improves kidney function in a CKD mouse model via miR-4516/SIAH3/PINK1 pathway. (**A**) miR-4516 expression level in renal cortex of healthy kidney control and CKD mice either treated with melatonin, or both melatonin and miR-4516 inhibitor, measured by qRT-PCR (*n* = 3). (**B**) Expression of SIAH3 and PINK1 in renal cortex of each groups. Protein expression level was quantified by densitometry and normalized to α-tubulin levels (*n* = 3). (**C**) IHC staining for SIAH3 in renal cortex of each mouse group. (**D**) H&E staining of renal cortex of each mouse group. (**E**,**F**) Measurement of blood urea nitrogen (**E**) and creatinine (**F**) level in serum of each mouse group (*n* = 5). The values represent mean ± SEM, * *p* < 0.05, ** *p* < 0.01 versus healthy kidney control; ^#^ *p* < 0.05, ^##^ *p* < 0.01 versus PBS; ^$^ *p* < 0.05, ^$$^ *p* < 0.01 versus melatonin. The α-tubulin was used as Western blot loading control for whole tissue lysates.

To further confirm our hypothesis that promotion of miR-4516/SIAH3/PINK1 signaling axis can help with the recovery from CKD-related mitochondrial dysregulation, we analyzed the effect of melatonin treatment in vivo. In response to melatonin treatment, CKD mice recovered from abnormal mitochondrial morphology (Figure 4A–C) and also from dysregulated mitochondrial dynamics (Figure 4D). Melatonin treatment also increased the colocalization of mitochondria with lysosome (Figure 4E) and autophagosome formation (Figure 4F). As expected, silencing miR-4516 abolished the effects of melatonin on mitochondrial regulation (Figure 4A–D). Corrective reprogramming of mitophagy is thought to have a protective effect against kidney injury and to reverse the progression of renal fibrosis. Based on our previous results showing that activation of miR-4516 mediated repression of SIAH3 can restore healthy mitochondrial dynamics and metabolism, we expected the melatonin-induced miR-4516 can improve the kidney function. To evaluate therapeutic efficacy of melatonin through miR-4516/SIAH3/PINK1 axis on kidney performance, we first confirmed that key proteins, SIAH3 and PINK1, were acting in accordance to in vitro data. PINK1 expression closely mirrored the expression of miR-4516, and SIAH3 was upregulated in the renal cortex of CKD mice (Figure 5A,B). Melatonin injection successfully increased the expression of miR-4516 and PINK1 translocation to mitochondria while suppressing the expression of SIAH3 (Figure 5A,B). Our histology data showed increased expression of SIAH3 in fibrotic renal tissues in CKD mice where miR-4516 was decreased (Figure 5C,D). Melatonin treatment effectively inhibited the expression of SIAH3 in renal cortex of CKD mice, and suppressed the fibrosis and glomerular hypertrophy in the renal cortex (Figure 5C,D). Again, silencing of miR-4516 reversed the therapeutic effect, confirming that the changes in kidney performance in each experimental CKD mice group can be attributed to the regulatory effects of miR-4516 (Figure 5A–D). We employed commonly used clinical measures for CKD, blood urea nitrogen (BUN), and creatinine in serum, to measure the changes in kidney performance in experimental mice groups. We observed significant decreases in both BUN and creatinine levels for melatonin-treated mice when compared with PBS control (Figure 5E,F), indicating markedly improved kidney function. Inhibiting the miR-4516 indeed reverses the improvements made in kidney functions as expected. Therefore, our data suggests that miR-4516/SIAH3/PINK1 mitophagy pathway can provide consistent explanations for the improvement we see for the pathologic features of in CKD mice with melatonin treatment. Collectively, these observations support the idea that targeting miR-4516 and its downstream effectors for mitochondrial regulation is a viable strategy for developing clinical treatments for fibrotic CKD.

## 4. Discussion

Mitophagy is one of mitochondrial quality control mechanisms that marks and eliminates excessive or defective mitochondria, which is crucial for maintaining healthy mitochondrial network [25]. Accumulating evidence suggests an impairment of PINK1/Parkin mitophagy pathway in acute and chronic kidney disease [7]. PINK1 and Parkin knockout mice shows more severe renal dysfunction in the nephrotoxin-induced CKD model [26] and the unilateral ureteral obstruction (UUO) model of renal fibrosis [13]. Other studies have confirmed the protective role of PINK1/parkin mitophagy in kidney diseases. PINK1/parkin signaling were found to decrease mitochondrial ROS (mtROS) and inflammasome activation in renal tubular epithelial cells [27], and to ameliorate fatty acid-induced apoptosis in diabetic kidney disease [28]. Similarly, a genetic study have demonstrated that overexpression of forkhead box class O1 (FOXO1) in rat kidney cortex, which enhanced PINK1/Parkin mitophagy, provided protective effect against high glucose-induced kidney injury [29]. Our observation of dysregulated PINK1/Parkin mitophagy in our CKD model is consistent with these findings, and reiterates the importance of this particular cellular process in renal pathology. 

As currently available treatment modalities for kidney fibrosis have a limited effect on stopping the progression of fibrotic CKD in most cases, there is a great need for identifying a better therapeutic target. For example, recent clinical trials of molecules inhibiting TGF-β and pirfenidone, a first in-class anti-fibrotic small molecule approved for idiopathic pulmonary fibrosis treatment, reported limited efficacy for kidney fibrosis [30]. Therefore, identifying a different target for renal fibrosis remains an important topic for further investigation. In light of this, dysregulated PINK1/Parkin mitophagy pathway is emerging as a promising target for many diseases, including CKD, as it seems that the process is responsive to therapeutic interventions with small molecules or miRNAs. For example, quercetin, a dietary flavonoid, can reduce RTEC senescence in renal fibrosis via SIRT1/PINK1/mitophagy axis [31], while liraglutide, a glucagon-like peptide-1 receptor agonist, can improve mitochondrial homeostasis in heart injury through the identical signaling axis [32]. In addition, PINK1/Parkin mitophagy has been shown to have upstream miRNA regulators like miR-155 [33], miR-181a [34], and miR-27a/b [35]. The results from our current study adds miR-4516 to the list, confirming miR-4516/SIAH3/PINK1 signaling as another pathway for targeting dysregulated mitophagy. 

Melatonin has been extensively investigated for its anti-fibrotic effects [15] and its regulatory role for mitophagy in heart [36], lung [37], liver [38], cancerous tissues [39], and kidney [16] has been confirmed in vivo, although different regulatory mechanisms have been reported for each organ or tissues. The anti-fibrotic effect of melatonin in kidney can be partly attributed to its ability to regulate miRNAs in different pathologies in different ways, which constitutes a novel kind of targeted therapy [40]. In addition, elucidating the regulatory mechanism of miRNA network downstream of melatonin can contribute to the development of miRNA-based therapy. For example, in our recently published study, we showed that melatonin-stimulated exosomes derived from kidney mesenchymal stem cell (MSC) are rich in miR-4516 and therefore capable of improve the impaired cellular function and proliferation of CKD-MSCs [41]. 

The relationship we observed between melatonin and miRNA network as pertains to mitophagy regulation hints at a link between miRNA expression and drug sensitivity. Previous studies of miRNA pharmacogenomics have shown that miRNAs can have the ability to determine the efficacy of the drug by downregulating the genes that are important for drug functions [42,43,44]. Likewise, our data showed that altering miR-4516 expression profile with inhibitor decreased the effects of melatonin, and caused upregulation of SIAH3 to suppress mitophagy. On a different note, we showed that shRNA-induced inhibition of SIAH3 mimics the effect of melatonin. Nonetheless, the rescue of mitochondrial autophagy and functions were incomplete, despite being statistically significant (Figure 5E,F). This encourages us to speculate the existence of a compensatory pathway downstream of melatonin, independent of miR-4516, for improving kidney function. In fact, melatonin has been reported to alleviate fibrosis by downregulating transforming TGF-β signaling in the heart tissues of diabetic mice [45], in cigarette smoke-exposed human mucoepidermoid cells [46], and in renal proximal tubular epithelial cells under high-glucose stress condition [47]. With TGF-β signaling having an important role in various kidney diseases [48], and with recent reports characterizing the crosstalk between miRNA and TGF-β pathways [49,50,51], it is plausible that TGF-β acts on melatonin-affected miRNA network to impact the fibrotic cellular processes, while being under the influence of melatonin. Further investigation into these interactions would help with fine-tune the miRNA-based strategies for treating chronic renal disease.

In addition to mitophagy, defective mitochondrial dynamics, including fission and fusion, were found to have an important role in the pathogenesis of acute and chronic kidney injuries and renal fibrosis [7]. Drp1, a small GTPase, regulates mitochondrial fission and their two major phosphorylation sites result in completely different downstream effects. Drp1 phosphorylation at serine 616 (p-Drp1(S616)) promotes fission [52], but its phosphorylation at serine 637 (p-Drp1(S637)) not only represses fission but leads to mitochondrial elongation via fusion [53]. Current model of CKD emphasizes excessive mitochondrial fragmentation, explaining that pathological upregulation of fission and suppression of fusion leads to progression of renal fibrosis [25]. While there exists a sound evidence for upregulation of p-Drp1(S616)-mediated fission in fibrotic renal fibroblasts in human and mice [54], we have observed a notable increase in pDrp1(S637) activity and mitofusion proteins in our CKD models. This result is consistent with our past published work with mitochondrial dynamic in CKD patient derived human MSCs [18] and supports the previously proposed idea that the effect and regulation of Drp1 for kidney disease is likely to be cell-context and stimulus dependent [55]. Therefore, additional research into Drp1 activity may help us understand different subcategories of renal fibrosis and their pathogenesis patterns.

Variety of miRNAs have been identified as important agents in pathophysiology of the injury and repair of kidney tissues and renal fibrosis in recent years [56], and at least five upregulated (miR-142-3p, miR-223-3p, miR-21-5p, miR-142-5p and miR-214-3p) and two down-regulated (miR-29c-3p and miR-200a-3p) miRNAs have been identified as potential biomarkers for kidney fibrosis by a recent meta-analysis study [57]. Although additional reports profiling miRNA expressions in renal fibrosis is rapidly accumulating, investigations into finding effective ways for targeting relevant miRNA-regulated subcellular processes has been trailing [58]. Here, our findings with in vitro and in vivo models suggest that miR-4516 is another important player in the dysregulation of mitochondrial autophagy in renal fibrosis of CKD, and miR-4516 promoted with melatonin can reliably restore mitophagy and kidney function. Our search for the downstream effector of miR-4516 in relation to its therapeutic effect has revealed the connection between miR-4516 and mitophagy through downregulation of SIAH. 

Although our previous study demonstrated that miR-4516 reduced dedifferentiation in renal fibrosis by regulating actin stress fiber [17], many researchers also think that renal fibrosis is induced by independent signaling, such as accumulation of mitochondrial dysfunction and dedifferentiation. Studies have shown the mitochondrial ROS increases to aggregating F-actin in yeast, and stress fiber induced mitochondria clustering and ROS generation in mitochondria [59,60]. These results support that a key factor play role in the decreasing actin stress fiber and accumulation of dysfunctional mitochondria. Our studies showed that miR-4516 is possible to reduce renal fibrosis by decreasing dedifferentiation and dysfunction mitochondria.

**Figure 6:**
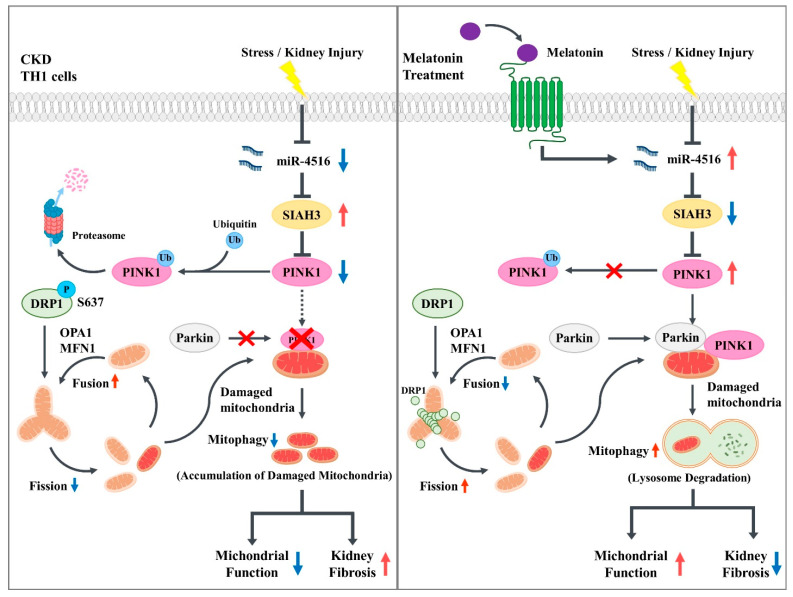
The schematic representation of miR-4516/SIAH3/PINK1 mitophagy pathway in CKD. Schematic representation of the proposed mechanisms by which melatonin-induced miR-4516 inhibits the progression of renal cortical fibrosis via PINK1/Parkin-mediated mitophagy signaling axis. Melatonin-induced miR-4516 suppresses the expression of SIAH3, which is overexpressed in CKD renal cortex, promotes PINK1/Parkin-mediated mitophagy, which reduces dysfunctional mitochondria, and improves kidney function.

In conclusion, the biochemical evidences we present establishes a pathway that connects the renal-protective effects of melatonin and its downstream miR-4516 regulation to PINK1/Parkin mitophagy (Figure 6). As there has not been a widely accepted cure for renal fibrosis and kidney injury patients yet, targeting micro RNA-mitophagy regulatory mechanism offers a promising strategy with the potential to reverse the progression of CKD and renal fibrosis. 

## Data Availability

Datasets were generated during the study. We endorsed MDPI Research Data Policies.

## Supplementary Materials

The following are available online at https://www.mdpi.com/article/10.3390/cells10071682/s1, Figure S1: Small hairpin-mediated knockdown of SIAH3 upregulates PINK1 to promote mitochondrial autophagy. Figure S2: Small hairpin-mediated knockdown of SIAH3 improved mitochondrial function by improved mitophagy in *p*-Cresol treated TH1 cells. Table S1: List of previously identified target genes of miR-4516 related to E3 ubiquitin protein ligase retrieved from mirDIP. Table S2: List of previously identified target genes of miR-4516 related to E3 ubiquitin protein ligase retrieved from miRDB. Table S3: List of previously identified target genes of miR-4516 related to E3 ubiquitin protein ligase retrieved from TargetScan.
