# Supplementary material for: Melatonin Treatment Improves Renal Fibrosis via miR-4516/SIAH3/PINK1 Axis
Source: Cells. 2021 Jul 3;10(7):1682. doi: 10.3390/cells10071682 (PMC8307213; doi:10.3390/cells10071682)
Supplement: Supplementary file 1 [file cells-10-01682-s001.zip › Supplementary Figure and figure legend.pdf]

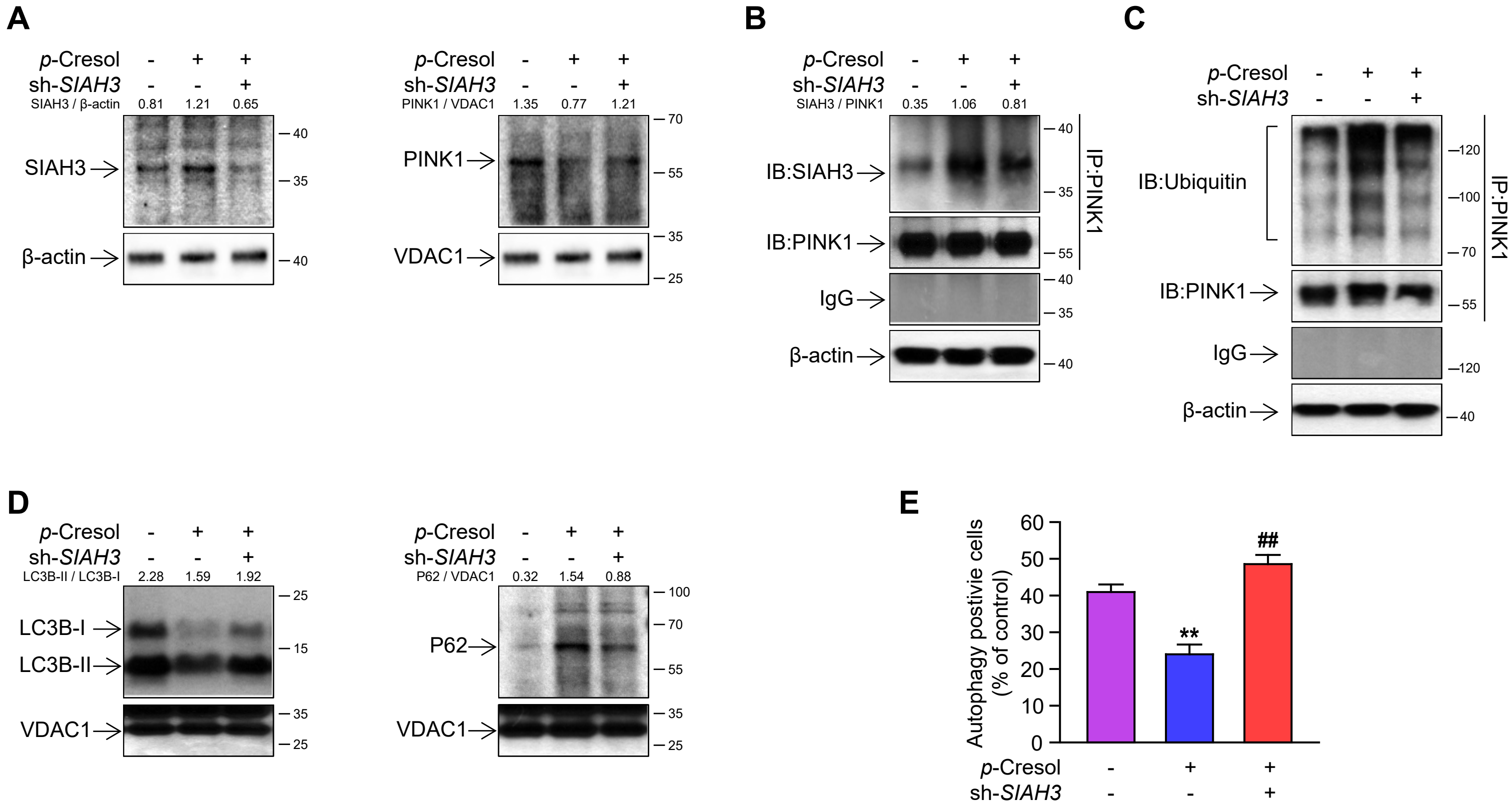

Supplemental figure 1

Supplementary Figure. 1. Small hairpin-mediated knockdown of SIAH3 upregulates PINK1 to promote mitochondrial autophagy. (A) Immunoblotting against SIAH3 and PINK1 for *p*-Cresol treated TH1 cells and sh-*SIAH3* treated TH1 cells under *p*-Cresol exposure compared with control. Protein expression level were quantified by densitometry normalized to β-actin or VDAC1 levels (n = 3). (B) Binding SIAH3 with PINK1 measured by immunoprecipitation for each group. Protein expression level were quantified by densitometry and normalized to PINK1 levels (n = 3). (C) Measurement of ubiquitination of PINK1 by SIAH3 using immunoprecipitation. (D) Expression of mitophagy markers LC3B and P62 for each group. Protein expression level was quantified by densitometry and normalized to VDAC1 levels (n = 3). (E) Detection of autophagy positive cells for each group. The values represent mean ± SEM, $^{**}P < 0.01$ versus control; $^{\#\#}P < 0.01$ versus *p*-Cresol exposure.

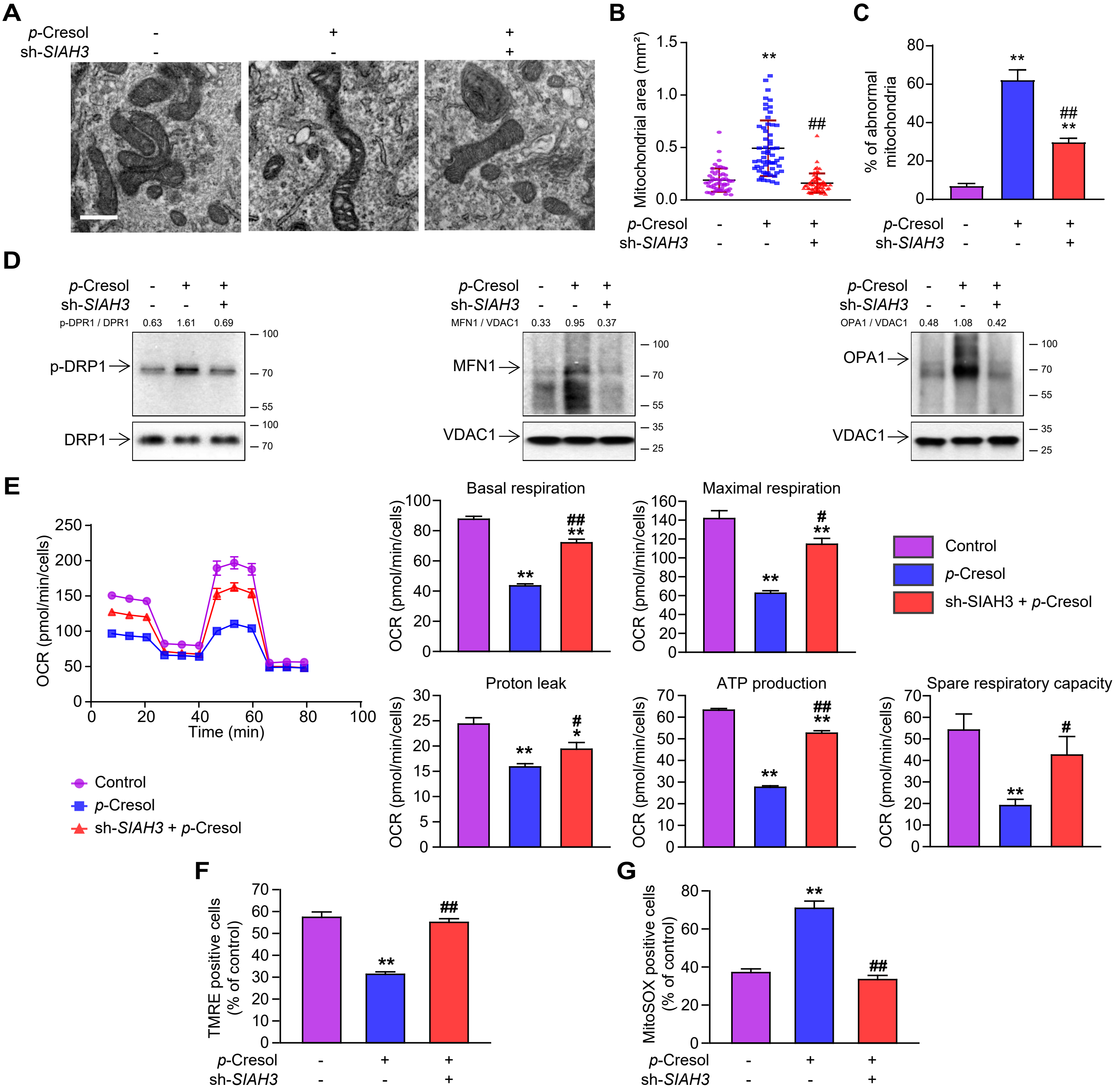

Supplemental Figure 2

Supplementary Figure. 2. Small hairpin-mediated knockdown of *SIAH3* improved mitochondrial function by improved mitophagy in *p*-Cresol treated TH1 cells. (A) Representative TEM images for *p*-Cresol treated TH1 cells or sh-*SIAH3* treated TH1 cells under *p*-Cresol exposure (scare bar =1 μm). (B, C) Measurement of mitochondrial area and number of abnormal mitochondria in each experimental group (n = 3). (D) The effects of melatonin on p-DRP1, DRP1, MFN1, and OPA1 were reversed with sh-*SIAH3* treatment. Protein expression level was detected using western blot, quantified by densitometry, and normalized to DRP1 or VDAC1 levels (n = 3) respectively. (E) Mitochondrial OCR of each group were obtained over time. Bioenergetics parameters calculated from OCR data by administrating oligomycin (20 min, 1 μM), FCCP (40 min, 0.75 μM), antimycin A (60 min, 1 μM), following the protocol described in the method section. The histogram graph represent data from one replicate experiment (n = 5). (F, G) Measurement of TMRE (F) and MitoSOX (G) positive cells for each group (n = 3). The values represent mean ± SEM, $^{*}P < 0.05$, $^{**}P < 0.01$ versus control; $^{\#}P < 0.05$, $^{\#\#}P < 0.01$ versus *p*-Cresol treated TH1 cells.
