# Supplementary material for: Melatonin Treatment Improves Renal Fibrosis via miR-4516/SIAH3/PINK1 Axis
Source: Cells. 2021 Jul 3;10(7):1682. doi: 10.3390/cells10071682 (PMC8307213; doi:10.3390/cells10071682)
Supplement: Supplementary file 1 [file cells-10-01682-s001.zip › Supplementary tables.pdf]

Supplementary table 1. List of previously identified target genes of miR-4516 related to E3 ubiquitin protein ligase retrieved from mirDIP.

Gene Symbol	Uniprot	Integrated Score	Number of Sources	Score Class
SIAH3	Q8IW03	0.314188206	9	High
PPP1R11	O60927	0.298769649	9	High
FBXW7	Q969H0	0.287065639	8	High
RLIM	Q9NVW2	0.27055211	9	High
ZFP91	Q96JP5	0.266212724	6	High
JADE2	Q9NQC1	0.255994518	9	High
TRIM41	Q8WV44	0.251925558	9	High
HECTD4	Q9Y4D8	0.215036497	9	High
RNF19A	Q9NV58	0.207907704	9	High
KCMF1	Q9P0J7	0.204549263	8	High
TRIM62	Q9BVG3	0.201592282	8	High
RNF114	Q9Y508	0.199811561	8	High
RNF220	Q5VTB9	0.197634454	8	High
RNF144B	Q7Z419	0.19727441	6	High
UBR7	Q8N806	0.19592164	9	High
TRIM13	O60858	0.190153657	9	High
RNF115	Q9Y4L5	0.183936719	9	High

Supplementary table 2. List of previously identified target genes of miR-4516 related to E3 ubiquitin protein ligase retrieved from miRDB.

Gene Symbol	Target Rank	Target Score	Gene name
SIAH3	41	94	Siah E3 ubiquitin protein ligase family member 3
RNF19A	315	80	Ring finger protein 19A, RBR E3 ubiquitin protein ligase
HECTD3	744	67	HECT domain E3 ubiquitin protein ligase 3
UBR7	960	62	Ubiquitin protein ligase E3 component n-recognin 7 (putative)
NEDD4L	1261	55	Neural precursor cell expressed, developmentally down-regulated 4-like, E3 ubiquitin protein ligase
UBR3	1383	53	Ubiquitin protein ligase E3 component n-recognin 3

Supplementary table 3. List of previously identified target genes of miR-4516 related to E3 ubiquitin protein ligase retrieved from TargetScan.

Gene Symbol	3P-seq tags + 5	Total sites	Gene name
CBL	309	3	Cbl proto-oncogene, E3 ubiquitin protein ligase
SIAH3	5	4	Siah E3 ubiquitin protein ligase family member 3
FBXW7	12	2	F-box and WD repeat domain containing 7, E3 ubiquitin protein ligase
HECTD2	1514	2	HECT domain containing E3 ubiquitin protein ligase 2
RNF19A	1674	1	Ring finger protein 19A, RBR E3 ubiquitin protein ligase
HECTD3	45	1	HECT domain containing E3 ubiquitin protein ligase 3
MUL1	812	1	Mitochondrial E3 ubiquitin protein ligase 1
MARCH7	537	1	Membrane-associated ring finger (C3HC4) 7, E3 ubiquitin protein ligase
RNF20	772	1	Ring finger protein 20, E3 ubiquitin protein ligase
MDM2	455	1	MDM2 oncogene, E3 ubiquitin protein ligase
RNF128	17	1	Ring finger protein 128, E3 ubiquitin protein ligase
PARK2	55	1	Parkin RBR E3 ubiquitin protein ligase
MIB1	897	1	Mindbomb E3 ubiquitin protein ligase 1
TRAF6	91	1	TNF receptor-associated factor 6, E3 ubiquitin protein ligase
PELI2	229	1	Pellino E3 ubiquitin protein ligase family member 2
BTRC	96	2	Beta-transducin repeat containing E3 ubiquitin protein ligase
RNF125	214	1	Ring finger protein 125, E3 ubiquitin protein ligase
NEDD4L	617	2	Neural precursor cell expressed, developmentally down-regulated 4-like, E3 ubiquitin protein ligase

Supplementary table 3. Continued.

Gene Symbol	3P-seq tags + 5	Total sites	Gene name
HECW1	11	2	HECT, C2 and WW domain containing E3 ubiquitin protein ligase 1
RNF8	301	2	Ring finger protein 8, E3 ubiquitin protein ligase
MARCH3	77	1	Membrane-associated ring finger (C3HC4) 3, E3 ubiquitin protein ligase
HECTD4	222	1	HECT domain containing E3 ubiquitin protein ligase 4
RFFL	670	2	Ring finger and FYVE-like domain containing E3 ubiquitin protein ligase
WWP1	161	1	WW domain containing E3 ubiquitin protein ligase 1
HACE1	191	2	HECT domain and ankyrin repeat containing E3 ubiquitin protein ligase 1
MARCH4	68	1	Membrane-associated ring finger (C3HC4) 4, E3 ubiquitin protein ligase
HECW2	28	1	HECT, C2 and WW domain containing E3 ubiquitin protein ligase 2
HUWE1	741	1	HECT, UBA and WWE domain containing 1, E3 ubiquitin protein ligase
